# Inducing stress, experiencing stress: Reflections and recommendations for the Trier Social Stress Test

**DOI:** 10.1016/j.cpnec.2026.100368

**Published:** 2026-07-16

**Authors:** Christiane Wesarg-Menzel, Veronika Engert

**Affiliations:** a Institute for Psychosocial Medicine, Jena University Hospital, Friedrich-Schiller University, Jena, Germany; b German Center for Mental Health (DZPG), Germany

**Keywords:** Trier social stress test, TSST, Stress, Empathy, Research ethics

## Abstract

Developed by Clemens Kirschbaum and colleagues in 1993, the Trier Social Stress Test (TSST) remains one of the most powerful and widely used experimental paradigms for inducing psychosocial stress. In this article, written in honour of Clemens Kirschbaum, we compiled anecdotal reports on researchers' experiences with the TSST. Fourty-six TSST researchers responded to our request, sharing insights into participant and panel member reactions, challenges in study implementation, and recommendations for the design of future stress induction studies. In sum, these accounts highlight that the TSST places a substantial burden not only on participants, but also, in a different way, on researchers serving as panel members. This observation raises intriguing questions for future research, such as whether imposing stress on others elicits a physiological stress response. Finally, for the readers’ amusement, the article recounts humorous anecdotes involving unusual participant performances and unexpected mishaps during experimental procedures.

## Highlights

• The Trier Social Stress Test (TSST) reliably induces psychosocial stress.
• Participant reactions range from severe distress to indifference.
• Panel members may also experience stress during the TSST.
• We provide best-practice guidelines for ethical TSST use.
• Key elements include careful screening, thoughtful task adaptation, and debriefing.

Cited more than 8000 times, the Trier Social Stress Test (TSST), developed by Clemens [1], is one of the most widely used experimental paradigms for inducing psychosocial stress. More than three decades after its introduction, the original protocol continues to be employed in laboratories worldwide. In the TSST, participants engage in a simulated job interview followed by a mental arithmetic task, both performed in the presence of an evaluative committee (also referred to as *panel*). Throughout the procedure, participants are positioned in front of a microphone and informed that their performance is video recorded for subsequent behavioral analysis.

The TSST reliably elicits a robust psychobiological stress response because it combines two key characteristics of stressful situations: social-evaluative threat and uncontrollability (Dickerson & Kemeny, 2004). Specifically, participants are exposed to the possibility of negative evaluation by others while having limited control over the outcome of the situation. As a result, the TSST induces increases in subjective stress, anxiety, and emotional insecurity, as well as elevations in cortisol and heart rate [2]. Its ecological validity is further supported by evidence demonstrating that cortisol responses to the TSST are significantly associated with cortisol responses to naturally occurring stressors, such as academic examinations [3]. Over the years, numerous adaptations of the TSST have been developed to accommodate different populations, research objectives, and practical constraints (for an overview of key reviews and protocol adaptations, see Box 1).

Box 1 **Common Adaptations of the Trier Social Stress Test (TSST)**

**Table:** 

Although the classic TSST consists of a mock job interview followed by a mental arithmetic task in front of an evaluative panel, numerous adaptations have been developed to address different populations, research questions, and practical constraints [4,5]. Examples include the TSST for children (TSST-C; [6]) and adolescents [7], which adapt instructions and evaluation procedures for younger participants, and the group TSST (TSST-G), which allows simultaneous testing of multiple participants while maintaining social-evaluative threat [8]. To study the formation and consolidation of memories associated with stressful experiences rather than solely stress reactivity itself, memory-focused variants have been applied, which embed standardized visual objects into the TSST environment and subsequently assess participants' memory for these objects [9,10]. To investigate stress contagion, the empathic TSST (E-TSST) requires participants to observe another person undergoing the TSST [11]. More recently, virtual reality TSSTs (V-TSSTs) have used computer-generated audiences and evaluators to increase standardization and reduce personnel requirements [[12], [13], [14]], while online implementations (TSST-OL) have demonstrated that key elements of social-evaluative threat can be delivered remotely [15]. These developments illustrate that the TSST is not merely a stress-induction procedure but a flexible experimental framework that can be tailored to investigate a broad range of questions concerning stress, cognition, social behavior, development, and psychopathology.
Researchers new to the paradigm may benefit from several key reviews. An introductory guide to conducting the TSST is provided by Labuschagne et al. [16]. The influential meta-analysis by Dickerson and Kemeny (2004) established social-evaluative threat and uncontrollability as central determinants of psychobiological stress responses. Goodman et al. [4] systematically examined how protocol variations influence cortisol responses, while Narvaez Linares et al. [17] provided a comprehensive review of methodological heterogeneity and reporting practices in TSST research. For virtual implementations, Helminen et al. [13,18] reviewed and meta-analyzed stress responses elicited by virtual TSST paradigms. Together, these reviews highlight both the robustness of the TSST and the importance of carefully considering protocol adaptations when comparing findings across studies.

Alt-text: Box 1

The road to success from the initial idea to the final, now “classic”, version of the TSST involved some piloting. As reported by a former colleague of Clemens Kirschbaum, early attempts at generating psychosocial stress involved paradigms in which participants were required to stand on a table and recite a poem or sing a song. Another pilot exposed participants to unpleasant noises that could only be turned off when the participant succeeded with a task. The task itself was, by design, unsolvable.

In this report, we bring together diverse experiences of the TSST research community, ranging from evaluations on the TSST's efficacy, over anecdotes on participant and panel member reactions, to shared advice on key aspects of TSST implementation. One specific section is devoted to funny anecdotes reported by the TSST community, profoundly evidencing that research has its lighter and enjoyable sides.

To compile reports, we contacted almost 300 researchers via e-mail who published TSST-related work between 2023 and 2025, identified through PubMed and personal contacts. Detailed information on data acquisition and processing, as well as anonymized versions of all reports, are provided in the Supplementary Material. We express our sincere thanks to the 46 contributors acknowledged herein, whose diverse experiences and recommendations are summarized in this paper. We hope that these community-shared insights will inform the future design of TSST studies, inspire further protocol adaptations (e.g., for use in vulnerable populations), and help address open questions through new empirical work. Finally, we hope that readers will find this paper an engaging and enjoyable read.

## 1 Expressions of praise for the TSST

Many TSST researchers began their anecdotal reports with a praise for the TSST's effectiveness and reliability in inducing psychosocial stress across diverse samples, including those with extensive job interview experience. Reports from multiple research groups support the strong cross-cultural validity of the TSST. Notably, even initially skeptical investigators observed clear cortisol responses in student samples they had expected to take the interview less seriously. The TSST's combination of ecological validity, standardization, and methodological adaptability has established it as the gold standard in laboratory stress research. Over time, the paradigm has even acquired its own nickname and is often referred to simply as “the TRIER”. For (former) students and researchers from Trier, however, the statement “We did the TRIER” reportedly carries a somewhat peculiar connotation.

The TSST is not only tremendously valued by researchers, but also acknowledged by participants, as illustrated by a recently published interview snip of a 52-year-old male participant: “And what was horrible for me was that test day. […] This stress test. I thought it was extremely hard, but also brilliantly good. I thought, what's going on? It was done professionally. It couldn't have been better. […] And I thought to myself, these people are either such narcissists or they were professional actors.” (from Ref. [19]).

## 2 Reactions during the TSST

### 2.1 Participant reactions

#### 2.1.1 Aversive reactions

When planning to administer the TSST, researchers should be prepared to encounter a broad range of behavioral responses. Several respondents to our survey reported that, consistent with the paradigm's intended psychosocial challenge, almost all of their participants exhibited some level of distress and discomfort—but completed the task without incident. Yet, more severe reactions can occur. In a small number of cases, participants displayed pronounced distress, including crying, freezing, nausea, profuse sweating, pallor, or fainting. In accordance with predefined safety criteria, these sessions were terminated immediately out of concern for participant well-being, and appropriate care and debriefing were provided. More commonly, participants ended their speech presentations earlier than planned due to running out of ideas; in most instances, they were able to resume speaking when prompted by the panel. Sometimes, however, participants remained silent for several minutes despite panel questions. Others exercised their right to withdraw, either after receiving the TSST instructions and before entering the task setting, or immediately prior to the mental arithmetic component of the task. One report described a participant disputing the correctness of the arithmetic feedback, while others became visibly angry and left the laboratory before completing the procedure. These behavioral responses were typically transient and resolved once the task was discontinued.

The strong impact of the TSST is further illustrated by an anecdote from a supervisor of a TSST study. While training several groups of students and therefore acting as a mock participant, the supervisor reported pronounced nervousness despite a 20-year age gap. Once the supervisor began to blush, the situation became genuinely embarrassing, highlighting the potency of the TSST even for experienced researchers and with full knowledge of the procedure.

While imagining participants undergoing the TSST naturally evokes empathy, the ethical implications become particularly salient when the procedure involves children and adolescents. Isolated reports described a nine-year-old who experienced urinary incontinence during the TSST, as well as a child who vomited after observing a peer undergo the task. Importantly, such incidents were extremely rare and have informed more stringent screening, monitoring, and adaptation procedures in subsequent research.

Researchers also reported unique response patterns in samples with psychopathology. For example, distress responses were particularly pronounced among individuals with elevated social anxiety. Among adolescents and adults with borderline personality disorder, administration of the TSST in a virtual reality setting elicited dissociative responses in some cases; importantly, these episodes were anticipated, monitored by trained personnel, and managed by medical staff without further complications. In a study involving children with autism, the speech task was adapted to focus on describing an ideal birthday party. While this modification aimed to reduce task difficulty, some participants reported that prior experiences of social exclusion—such as having no peers attend past birthday celebrations—rendered the task emotionally more challenging.

Overall, these reports underscore the importance of general awareness regarding the TSST's potential effects and the necessity of careful implementation, particularly when working with vulnerable populations. A broader discussion of differential vulnerability is provided by Ghabrial [20], who highlights how psychosocial stress paradigms may affect lesbian, gay, bisexual, transgender, queer, asexual, and other sexual and gender minority individuals in distinct ways, particularly in the context of chronic minority stress and prior experiences of stigmatization. Rather than constituting a contraindication for the use of laboratory stress paradigms, these considerations emphasize the need for careful screening, thoughtful task adaptation, and thorough debriefing.

Taken together, these experiences illustrate that while the TSST reliably induces psychosocial stress, its ethical application rests on active distress monitoring, clearly defined termination criteria, adaptive implementation, and respect for participant autonomy. Experienced researchers consistently view these safeguards not as optional add-ons, but as integral components of responsible task use. We summarize best practice recommendations for the ethical implementation of the TSST in Box 2. A more detailed discussion of ethical considerations is provided in Broeder et al. [21], who published consensus guidelines on laboratory stress research in psychiatric and neurological populations.

Box 2 **Ethical use of the Trier Social Stress Test (TSST)**

**Table:** 

The ethical use of the TSST relies on anticipating and actively managing participant distress rather than avoiding stress induction per se. Best practice includes thorough pre-screening for vulnerability factors (e.g., severe social anxiety, trauma history, or clinical conditions associated with dissociation), clear communication of participants' right to withdraw at any time without penalty, and continuous monitoring of behavioral and physiological responses during task administration. Researchers should define explicit termination criteria in advance (e.g., fainting, severe distress, dissociation) and ensure that trained personnel are present to intervene when necessary. Task adaptations may be warranted for specific populations (e.g., children or clinical samples), and careful debriefing should be conducted to contextualize the stress experience and restore participants' sense of control. Collectively, experienced TSST researchers view these measures as integral components of responsible implementation, ensuring that the paradigm's scientific value is balanced with participant safety and autonomy.

Alt-text: Box 2

#### 2.1.2 Non-aversive reactions

As is common in research on individual differences, some participants occasionally exhibited non-aversive rather than distress reactions to the TSST, at least on the level of behavior and self-report. While some individuals reported little discomfort and indicated that they did not even feel nervous, others appeared to actively enjoy the stress testing experience. Also, and irrespective of the intensity of felt stress, many participants ultimately expressed appreciation for the opportunity to participate in a challenging experiment and reported a sense of satisfaction upon its successful completion. Notably, one researcher observed that the TSST was largely ineffective in eliciting stress responses among military veterans, and this was true both subjectively and in terms of cortisol reactivity. This special population seemed to be generally unfazed by the procedure or even described it as enjoyable.

### 2.2 Panel reactions

The TSST is not only effective at inducing stress in participants but can also elicit stress responses in panel members. This phenomenon was frequently reported by the surveyed TSST researchers, who recalled research assistants remarking half-jokingly, “You should be sampling my cortisol right now!”. One distinguished professor, who had conducted numerous TSSTs as a student assistant, noted that he never habituated to the procedure. Across all sessions, he consistently felt uneasy and stressed, empathizing deeply with the participants. Unsurprisingly, he now appreciates being able to remain in the background while a new generation of young researchers gains first-hand experience administering the TSST. The stress experienced by TSST panel members may also have practical implications. As one researcher noted, finding research assistants willing to take on this demanding role can itself become a considerable challenge.

In two reports, the TSST was described as stressful not only for participants and panel members but also for the entire research team conducting the testing session. Coordinating the timing of each component, maintaining the integrity of the scenario, and managing unexpected participant behaviors posed considerable challenges for the team. Staff members often found themselves on high alert, taking care to avoid accidental encounters with participants or quickly adapting to unforeseen situations. In one study, a participant reportedly ran back and forth along the hallway, exclaiming, “Hey, young lady, there is blood spraying out of my arm!”, while inadvertently leaving traces of blood behind. The take-home message from this research group was the importance of remaining calm as the lead experimenter. Overall, despite these unforeseen situations and high demands, TSST researchers emphasized that the effort is well worth it, given the rich and ecologically valid information the TSST provides about stress reactivity.

As was occasionally observed among participants, some panel members appeared particularly engaged in their role during the stress-induction procedure. One report described a student assistant who had served as an officer in the German Armed Forces before starting his studies and seemed to be highly at ease in his role as a panel member (perhaps a little too much). The researchers were seriously concerned that their participants’ cortisol responses in the TSST might disappear once the former officer left the study; fortunately, this concern turned out to be unfounded.

Another report was about a female student assistant who thrived in administering the TSST and took pride in her ability to elicit strong stress responses. She reported informally tracking particularly intense participant reactions during sessions in which she served on the panel and occasionally shared these experiences with the research team as illustrations of her impact in the task. Surprisingly, her attitude toward running TSST proved contagious. Over time, other research assistants began to enjoy running the stress sessions as well and, rather than dreading intense participant responses, they came to hope for them.

Taken together, these anecdotes suggest that serving as a TSST panel member is experienced as stressful by most individuals. However, some may find the role enjoyable, perhaps owing to individual differences in personality, including traits such as extraversion, psychopathy, and Machiavellianism [22]. Importantly, enjoyment of the role does not appear to be a prerequisite for effective stress induction.

## 3 Researcher reflections and colloquial wisdom

### 3.1 Observations and reflections

#### 3.1.1 Participant behavior

The reports sent by TSST researchers demonstrate that this community is not only deeply interested in the biological underpinnings of stress, but also attentive to behavioral signs at both macro- and micro-levels. One researcher reported that during the TSST, nearly every participant had a hallmark fidgety motion that they would perform once sufficiently stressed. This was usually a tapping of the foot, swaying of the body, or twisting of hair. It was interesting to note that although everyone experiences stress in different ways and with varying intensity, these fidgeting motions were largely consistent across participants. Another interesting observation was that nearly every participant presented efficiency in one aspect of the TSST—but struggled with the other. For example, if a participant spoke confidently and without worry during the speech portion, they usually experienced difficulties with the mental arithmetic and vice versa. This quirk was particularly evident in cases where participants expended most of their energy in delivering the speech, only to be informed afterward that they were still required to complete the arithmetic task. These participants appeared especially stressed, likely because they believed that their struggles would end once the speech was accomplished. The resulting violation of expectations was likely a contributing factor to their heightened stress response.

In another account, the video camera was described as the most discomfort-inducing element of the stress-induction procedure. This observation was interpreted as reflecting heightened perceptions of social evaluation and self-consciousness elicited by being recorded—an experience that differs from most everyday contexts, in which constant observation is fairly uncommon. Consequently, concerns were raised regarding the generalizability of findings, as participants’ responses to the TSST may be amplified compared to real-life public speaking situations.

Not all participants may engage equally with experimental procedures. In a study involving children, two nine-year-old boys filled their preparation period exclusively with “yo mama” jokes, leaving the target child completely unprepared once the speech began. Further, in a study with adolescents, reduced levels of collaboration were occasionally observed, potentially reflecting developmental characteristics associated with this life stage.

#### 3.1.2 Challenges in study implementation

As is typical in experimental research, a variety of practical and methodological challenges can arise. The following section summarizes the diverse challenges experienced and shared by TSST researchers, including the resource-intensive nature of the TSST and partially automated implementations as a potential solution, the development of a non-stressful control condition, staff coordination, cultural factors such as napping practices influencing cortisol assessments, hormonal sampling procedures, and challenges associated with the COVID-19 pandemic.

Several researchers remarked that the TSST is highly demanding in terms of time and resources. For this reason, some have implemented partially automated versions of the task. In one such adaptation [23], panel members' instructions were pre-recorded and presented to participants via an online video call. To enhance credibility of the panel's supposed presence and to answer arising questions, the experimenter joined the video call in person. The researchers reported technical challenges in maintaining the reliability of the experimental procedure, as software updates occasionally disrupted the programmed setup. In some cases, these disruptions led to the loss of participant data. Ultimately, although the partially automated version remained time- and resource-intensive, it was perceived as a reasonable compromise between available resources and effective stress induction. In fact, adaptations of the TSST for virtual settings have been documented across a wide range of publications (e.g., Ref. [[24], [25], [26]]). Meta-analytic evidence suggests that virtual forms are as effective as the traditional TSST in eliciting self-reported and physiological stress responses [18].

The development of a good (i.e., non-stressful) control condition was further noted as a challenge. In a virtual reality study with borderline personality disorder patients and healthy controls [27], the TSST control condition was adopted from the friendly TSST [9,10]. In this version, participants talk freely about their life and career aspirations in front of a casually dressed committee that reacts in a friendly way by nodding and smiling to give participants a feeling of safety [9,10]. In the study of Ref. [27], to further reduce the threat of the procedure, participants were informed that committee members were new to their job and might make mistakes themselves. Moreover, committee members did not request restarts after arithmetic errors. Nevertheless, this adapted version caused a relatively high level of stress in participants, irrespective of the presence of borderline symptoms.

In longitudinal studies, including those employing ecological momentary assessment designs, researchers reported challenges arising from the use of students as both panel members and participants. Throughout data collection, efforts were made to minimize contact between these two groups. Nevertheless, when student panel members occasionally encountered peers in shared university spaces, they attempted to avoid these interactions, that is, they literally hid. Such precautions were deemed necessary to preserve the stress-inducing potential of the panel, which could be diminished if panel members were already familiar to participants or became identifiable as fellow students during data collection.

Given the international nature of the TSST research community, an interesting observation emerged regarding cultural differences in experimental implementation. One researcher reported conducting the TSST in Southwest China, where the recommended afternoon administration of the task [4,16] coincided with a strong local tradition of post-lunch napping. Because prior research indicates that napping can influence basal cortisol levels [28], concerns were raised about potential interference with cortisol reactivity during the TSST. These concerns ultimately proofed unfounded, however, as robust cortisol stress responses were observed even in this population of habitual nappers.

The collection of salivary cortisol was identified as a disruption to the experimental procedure. The use of wearable devices capable of collecting and analyzing cortisol from sweat was suggested as a more undisturbed alternative. Another reported methodological challenge pertained to the assessment of electrocardiograms (ECG) in children, who may move frequently or have difficulty remaining still, potentially introducing artifacts into the signal.

Many researchers reported the Covid-19 situation to have been a major challenge, since it suddenly became necessary to maintain physical distancing while still conducting the TSST in person. In response, various modifications to the experimental setup were implemented, reflecting considerable methodological creativity. These included alternative table and camera arrangements, the use of hygiene screens and clear face shields, and reducing the panel to a single member administering the task. Notably, one researcher reported that panel members wearing medical face masks prevented successful stress induction. This finding suggests that visible facial cues—particularly the absence of smiling—may play an important role in TSST efficacy.

### 3.2 Recommendations

Several researchers who responded to our request shared recommendations addressing different stages of TSST research, ranging from study planning to participant debriefing. Accordingly, the recommendations presented here reflect issues currently considered relevant, though they are not intended to be exhaustive. A summary of key takeaways relevant for study planning and implementation is provided in Table 1.

**Table 1:** Recommendations for Trier Social Stress Test (TSST) planning and implementation.

Recommendation	Concrete Action
**Preparation**	
Use a multi-system assessment approach	• Include measures from multiple stress-response systems rather than relying on a single outcome
Conduct outcome-specific power analyses	• Calculate sample size separately for each primary outcome • Account for differences between psychological and physiological stress responses
Screen for medication effects	• Exclude participants taking medications that affect stress biomarkers or record medication use as covariates • Pay particular attention to ADHD and ASD medications affecting cardiac activity
Ensure participant safety	• Provide immediate access to a place where participants can lie down • Remove environmental hazards • Keep a first aid kit readily available
Plan for unexpected events	• Establish protocols for emergencies and disruptions in advance • Decide when confederates should remain in role versus break character
**Experimental Set-up**	
Maximize standardization	• Standardize laboratory conditions, experimenter attire, behavior, and procedures
Carefully structure recovery periods	• Use standardized activities (e.g., questionnaires) during rest periods • Avoid questionnaires that may themselves induce stress
Assess pre-experimental stressors	• Ask participants about unusual events before testing (e.g., accidents, major stressors)
Consider language proficiency	• Ensure participants can comfortably perform tasks in the testing language
Promote comfort during ECG placement	• Consider same-sex experimenters, especially for adolescents • Explain procedures thoroughly and respectfully
Modify tasks for familiar participants	• Change interview topics and arithmetic sequences if participants are familiar with the TSST • Evaluate whether the modified task remained stressful afterward
Adjust task difficulty when needed	• Increase difficulty only when justified (e.g., additional social-evaluative elements) • Reduce difficulty for vulnerable populations
**Panel Member Conduct**	
Maintain a neutral demeanor	• Speak slowly and clearly • Maintain eye contact with participants • Avoid nodding, smiling, or showing approval (“think: robot”)
Use silence strategically	• Allow pauses before prompting participants to continue • Repeat prompts if necessary without reducing pressure
Manage emotional reactions	• Take notes to maintain neutrality • Use discreet strategies to suppress laughter • Avoid eye contact between panel members
Select committee members carefully	• Avoid pairing individuals who are likely to make each other laugh
Address panel member discomfort	• Remind staff that participant stress is temporary and followed by debriefing
**Termination and Debriefing**	
Establish stopping criteria	• Define clear rules for terminating sessions due to severe distress • Monitor participants continuously for signs of excessive stress
Respect participant autonomy	• Offer participants the option to withdraw or take a break • Resume only with explicit consent
Conduct comprehensive debriefing	• Explain the mock nature of the TSST • Clarify that panel members were role-playing • Explain the purpose of the stress induction
Facilitate post-session interaction	• Offer participants the opportunity to meet panel members after the task
Reduce long-term negative associations	• Ensure debriefing is thorough and emotionally supportive • Address lingering questions or concerns before participants leave the laboratory

#### 3.2.1 Preparation

Once a study idea is sufficiently concrete, researchers must address the often demanding task of conducting a power analysis. Despite the challenges associated with accurately estimating sample size, this step is essential for both statistical and ethical reasons. Given that stress response magnitudes vary across outcome measures and that psychological and physiological systems are only loosely coupled (see Ref. [29]), researchers are advised to carefully determine the sample size required for each specific outcome and to adopt a comprehensive, multi-system approach when designing TSST studies.

In addition, awareness was raised regarding medications that may affect biological measures of stress reactivity. Such medications should either inform exclusion criteria or be documented as potential covariates. For instance, substances commonly prescribed for autism spectrum disorder or attention-deficit/hyperactivity disorder can influence cardiac activity and, consequently, ECG parameters.

With respect to the testing environment, it was repeatedly recommended to ensure that participants have access to a space where they can lie down immediately if necessary (e.g., in case of dizziness). Researchers should also be mindful of potential hazards in the immediate surroundings. For instance, one report described a participant who lost consciousness during a blood draw and struck their head on a heater. In all cases, having a first aid kit readily available is indispensable.

It is also advisable to discuss in advance how unexpected situations should be handled during the TSST, including whether confederates should remain in role and to what extent deviations are appropriate under specific circumstances. This recommendation stems from an incident in which a building-wide alarm went off at the end of a testing session. Although the panel members were as surprised as the participant, they attempted to stay in character and instructed the participant to return to the experimenter. While the panel had prepared for anticipated challenges, such as participants becoming tearful, the unexpected disruption proved more difficult to manage while maintaining composure and procedural consistency.

#### 3.2.2 Experimental set-up

Standardization was emphasized as a critical aspect of TSST implementation, encompassing the laboratory environment, experimenters’ attire and behavior, and procedural details. During the post-TSST resting period, it is advisable to include activities that can be administered in a standardized manner. To make effective use of the recovery period, some researchers recommend administering questionnaires. While this can be a productive way to occupy participants and represents a relatively controlled activity (as opposed to smartphone use), it needs to be noted that, for some individuals, reflecting on their stress load may itself be stressful. This may be particularly the case when questionnaires address topics such as social support or relationship quality, depending on the study focus, and could therefore confound stress assessments.

It was further recommended asking participants about any unusual events prior to the start of the experiment. One researcher reported extremely high pre-TSST cortisol levels in a pair of twins who later disclosed that they had been involved in a car accident about 1 h before their appointment.

Participants’ native language was also brought forward as an important point to consider. One report was about a participant who, although fluent in the testing language, struggled with the arithmetic task. The participant began to confuse certain numbers, becoming increasingly frustrated, and emotional.

When the experimental setup involves placing electrodes on participants’ chests to assess an ECG, particular sensitivity is required to ensure participant comfort. For example, adolescent female participants have occasionally reported embarrassment in the presence of male experimenters during electrode placement. This occurred despite thorough explanations of the procedure, the absence of any need to remove clothing, and electrode placement well away from intimate body regions. It illustrates that discomfort may still arise despite careful preparation, particularly among adolescent participants, and suggests that recruiting same-sex experimenters may be advisable in such contexts. A related report was about placing electrodes on autistic participants, who often exhibit heightened sensitivity to tactile stimuli. In this population, electrode placement needed to be performed rapidly while engaging participants in conversations about their personal interests as a form of distraction. Given their frequent curiosity about technology, it was also essential to provide clear and detailed explanations of each step involved in electrode placement and ECG recording.

Additional recommendations concerned adaptations of TSST task instructions. If a participant is already familiar with the task—for example, through a friend or classmate who previously participated—it is necessary to modify both the interview and mental arithmetic components to preserve the validity and fairness of the procedure. For instance, the interview may cover alternative topics, and the mental arithmetic task may involve subtraction based on different numbers. In such cases, it is important to assess afterward whether the participant experienced the TSST as stressful.

When researchers wish to increase task difficulty, one suggestion involved having participants perform the task while wearing a childish red plastic raincoat. Another recommendation, specific to Swiss samples, was to require participants to count backwards in High German, although concerns were humorously raised about the potential impact on cross-national friendship. Conversely, reducing task difficulty may be appropriate when applying the TSST in vulnerable populations (see Ref. [20]).

#### 3.2.3 Panel member conduct

Further guidance was offered for panel members. To ensure a professional and standardized demeanor, it was recommended that they speak slowly and clearly, maintain eye contact, and avoid nodding in agreement—summarized by one TSST researcher as “think: robot.” If participants pause or stop during the interview, panel members are advised to wait briefly before prompting them to continue, even if such prompts need to be repeated multiple times. Although these pauses may feel uncomfortable, they effectively induce stress; accordingly, tolerating periods of silence is considered best practice.

To help maintain a neutral demeanor, several strategies were proposed, including taking notes on participants’ behavior or specific aspects of the interview. If a smile or giggle cannot be suppressed, it may be helpful to briefly pretend to sneeze or adjust something near the upper lip, thereby discreetly covering the mouth with the hand. Regardless of the strategy employed, panel members are strongly advised to avoid making eye contact with one another, as this may trigger contagious laughter.

In this context, the composition of the committee should also be carefully considered. Pairing individuals who tend to elicit laughter in one another may be suboptimal. If such a pairing cannot be avoided, repeatedly suppressing laughter may even lead to physical discomfort, particularly in habituation studies involving large numbers of consecutive testing sessions.

Finally, committee members may experience feelings of guilt or discomfort when observing participants’ stress. One researcher reported that, in such situations, it can be helpful to remind oneself that the stressful phase is brief, that participants will be thoroughly debriefed afterward, and to remain focused on the task at hand.

#### 3.2.4 Termination of the experiment and debriefing

Our respondents repeatedly mentioned that clear rules are needed on when to terminate the experiment. Investigators should remain vigilant and terminate the experiment promptly upon recognizing signs of severe distress to prevent potential safety incidents. In such cases, it may be advisable to ask participants whether they wish to withdraw from the experiment or whether a brief pause is sufficient, with the procedure resuming only after the participant provides consent to continue.

Considerable attention should also be given to the debriefing of participants following the TSST, to ensure that they leave the laboratory in a positive emotional state. The debriefing should include a clear explanation of the mock nature of the setting, emphasizing that the panel was role-playing and that no video recording took place (if applicable). The experimenter should also explain why this procedure is typically experienced as stressful and offer participants the opportunity to meet the panel members. Such interactions may be stress-relieving not only for participants but also for panel members, as they allow space for clarification, reflection, apology, or mutual acknowledgment of the potentially stressful nature of the TSST.

The relevance of thorough debriefing is further illustrated by an anecdotal report from a former student confederate. Two years after serving as a panel member in several TSST sessions, the student attended a university seminar, where she noticed a classmate watching her from the corner of her eye. As the seminar pertained to emotional memory, the confederate shared her experience with the TSST. This prompted the classmate—who had previously participated in the TSST—to realize where they had met. The classmate explained that she had felt hesitant to approach the confederate, feeling an unidentifiable discomfort when she saw or heard her speak, even after all that time. This unease subsided once the two became better acquainted. The account suggests that associations formed during the TSST may persist over extended periods and underscores the importance of comprehensive debriefing to mitigate lingering emotional responses.

### 3.3 Colloquial wisdom

After years of experience, certain insights naturally emerge. Several important maxims were shared with us by the contacted researchers. Many would likely agree that the first participant is always like the first pancake—never quite perfect. Likewise, we all may have experienced the inevitable failure of an overly complex study set-up: when too many elements (e.g., sensors, questionnaires, hardware adjustments) are introduced at once, something is bound to go wrong.

Yet humor is essential. For many researchers, the TSST has been a central component of their doctoral training, often involving years of participant testing, troubleshooting protocols, and dealing with the inevitable uncertainties of experimental research. Without a sense of humor, navigating these challenges would be unimaginable. In the spirit of mental hygiene, why not calling the data a “dopey cow”? We cannot disclose the person responsible for this statement, but what we can uncover is a wisdom attributed to Clemens Kirschbaum himself, made after several TSST experiments: never postulate a directed hypothesis, as the results often turn out the opposite of what was expected. We are confident that this remark was intended humorously rather than as a serious methodological recommendation.

## 4 Funny anecdotes

Across laboratories, years of data collection with the TSST have generated not only robust psychophysiological findings but also a rich archive of unexpected, humorous, and sometimes bewildering participant behaviors. Below, we summarize several of the reported experiences, illustrating the diversity of human aspirations and responses to psychosocial stress. While these anecdotes are shared partly for amusement, they also serve as a reminder that participant behavior can be unpredictable and that effective panel members must occasionally respond with flexibility and spontaneity while maintaining the integrity of the protocol.

### 4.1 Unusual job applications

Given the open-choice format of the commonly used job interview in the TSST, a series of unexpected job applications were encountered by panel members. One participant applied for a clown school, and the demonstration of his skills as a future clown led to uncontrollable laughter among the committee. Surprisingly, a post-hoc check of the participant's cortisol levels revealed a marked increase despite the hilarious interaction. In other sessions, an opera singer warmed up with vocal scales during preparation, and a musician delivered an exaggerated, theatrical performance that almost broke the committee's composure. Another participant applied as a gigolo, prompting the panel to end the interview early due to inappropriate content. Finally, a participant described their ambition to become a sexual therapist and elaborated at length on relevant professional experiences.

A separate line of anecdotes comes from training sessions, where student research assistants simulated unusual professions to challenge the team, such as in the job application as a beekeeper who communicates with bees via dance.

### 4.2 Surprising performances

Despite a highly structured setting, participants' behavior can come at a surprise to committee members. To lighten the mood in the job interview part of the TSST, one participant began telling jokes. Another participant already entered the TSST with a bright, enthusiastic smile and remarkable energy. He immediately launched into a passionate monologue describing why he was the ideal candidate to become a physician, recounting his childhood aspirations and his strong vocational commitment to caring for others. However, after approximately 2 min, he reached his conclusion. Unexpectedly, he then adopted a static, almost mannequin-like pose, turned directly toward the camera, and proceeded to ignore the committee entirely. When reminded by the committee that time remained to continue his speech, he responded with a statement akin to, “Thank you, I'm fine,” and remained motionless, smiling directly at the camera. After the prompt was repeated and clarified—emphasizing that he was *required* to speak for the full 5 min—he again declined, politely but firmly, maintaining his fixed posture and expression. Throughout this period, he appeared entirely indifferent to the evaluative presence of the committee.

Another participant used the speech period not to describe a dream job but to present an elaborate philosophical reflection on how their current life situation existed in a box and how they wanted to break free from it. They constantly used hand gestures to draw a box in the air and motion as if they were destroying and rebuilding a new box. With a lot of confidence and joy, the participant did not run out of things to say in the allotted 5 min and had to be alerted and stopped at the time limit. While most participants exhibit proficiency in only one of either the speech or arithmetic portion, this participant continued their sprint by not making a single mistake while completing the challenging arithmetic task.

Although the mathematical part of the TSST is designed with a high level of difficulty, also other researchers reported having encountered participants with no difficulties in this task. In one twin study, both twins completed the backward counting task in just under 4 min without any error —they were monozygotic of course.

Other participants used environmental resources in unconventional ways. One individual used saliva to “write” on the one-way mirror as an aid during mental arithmetic, prompting an extended sanitizing session afterward. Another stepped away from the microphone mid-instruction to polish their glasses at the room's sink, while a very tall participant simply lifted the entire microphone stand to bring it to his level.

### 4.3 When the committee can't refrain from laughing

In the following situations, committee members reported difficulty maintaining a neutral demeanor. For instance, one participant applied to the German railway company and highlighted “being always on time” as a key strength of the organization. Another participant ended the speech task after just 1 min, remained silent for several seconds, and then added, “Also, I'm very efficient.” In a further case, a participant verbalized her repeated failures during the mental arithmetic task at a metacognitive level, stating something like “2009 … Yeah, damn, I already screwed that up just now—let's see if it works this time: 1993! Phew, OK, next attempt: 2043, 2026, 2009. So, now I'm back at this difficult part again … 1994?”.

### 4.4 Mishaps

As with any experiment, things can occasionally go wrong. In one TSST session, a participant noted that the Salivette used for cortisol collection felt wet. It turned out that it was the one given to the previous participant. Another participant repeatedly failed to reinsert the cotton swab into the Salivette, likely reflecting elevated stress levels.

## 5 Open questions

Although the effects of the TSST on participants have been extensively examined, several TSST researchers have raised questions regarding its impact on panel members. In light of frequent self-reports of distress among individuals serving as panelists, the hypothesis has emerged that panel members may also exhibit significant increases in cortisol. An answer to this question is provided in Buchanan et al. [30], who showed that on average, panel members do show significant cortisol increases in response to the procedure, whereas salivary alpha-amylase seems to increase only marginally. Given that stress transmission can occur between participants and panel members [30] or passive observers [11] during the TSST, future studies could further examine the factors underlying the emergence of behavioral, physiological, or neural synchrony. Such work may offer a novel perspective on the dynamics of social-evaluative stress.

Another suggestion made by respondents was to consider whether stress responses in panel members vary across cultural contexts. Given that reports of distress among panelists have originated from diverse cultural backgrounds, intuitively, we would not expect systematic cultural differences. Nevertheless, we strongly encourage cross-cultural collaborative research to empirically examine the issue.

An area of research that is rapidly advancing concerns the prediction of stress responses using multimodal markers. Studies employing the TSST have shown that stress reactivity can be predicted from movement dynamics [31,32], speech features [33], and gaze behavior [34]. So far, most studies have examined these modalities separately. Consequently, it remains unclear whether integrating multiple markers enhances the prediction of stress reactivity and whether such multimodal profiles are associated with vulnerability to stress in daily life.

Future research could further advance developmental perspectives on psychosocial stress reactivity by integrating TSST-based research with age-appropriate stress paradigms across the lifespan. Although the TSST and its adaptations have substantially enhanced our understanding of stress responses in older children, adolescents, and adults, the paradigm is less suitable for very young children, who may be more sensitive to other forms of stress than to social-evaluative threat. A particularly promising direction is the development of a coordinated set of age-appropriate stress paradigms spanning early childhood through adulthood. Such an approach would enable researchers to examine the continuity and change of stress responses across development, as well as the individual and environmental factors that shape these trajectories. Ultimately, this could provide a more comprehensive understanding of the developmental origins and lifespan stability of individual differences in stress reactivity.

Looking ahead, future research should continue refining the TSST while preserving the strengths that have made it the gold standard for laboratory stress induction. Particular attention could be directed toward reducing the paradigm's substantial resource demands through automated or hybrid implementations, improving understanding of the mechanisms underlying social-evaluative threat, and examining cultural and developmental factors that may influence stress responses. Furthermore, little is known about the experiences and characteristics of TSST panel members themselves, despite their central role in the procedure. Addressing these questions may not only improve the practicality and ethical implementation of the TSST but also deepen our understanding of the social processes that underlie human stress responses.

## 6 Words of honour

On behalf of the TSST research community, we express our sincere gratitude to Clemens Kirschbaum for his pioneering and foundational contributions to stress research. Among his many achievements as a researcher, collaborator, mentor, and lecturer, the development and dissemination of the TSST stands out as one of the most enduring. Across multiple continents, his direct involvement in the analysis of cortisol samples has been deeply valued, particularly his meticulousness and his proactive engagement when cortisol patterns deviated from expectations. Many researchers spoke of their collaboration with him as a true privilege. In at least one stress laboratory, a room in which TSST sessions are conducted has been named the “Kirschbaum room”, alongside rooms named “Selye”, “Cannon”, and “McEwen”. This symbolic recognition reflects the profound and lasting impact of Clemens Kirschbaum's lifetime achievements and his standing as one of the leading figures in psychoneuroendocrinological research.

## 7 Conclusion

While the TSST is a highly standardized and validated psychosocial stress induction procedure, the anecdotes summarized in this article illuminate the variability and creativity of human behavior under evaluative threat. Across studies, participants have turned the TSST into a stage for humor, improvisation, existential reflection, or complete withdrawal—challenging researchers to maintain neutrality while providing deep insight into the diversity of stress responses and coping therewith. On the other hand, the reports highlight that the TSST research community is composed neither of narcissists nor professional actors, but of individuals who strive to conduct rigorous research and who themselves experience the stressful burden associated with inducing stress in others.

## Declaration of generative AI and AI-assisted technologies in the manuscript preparation process

During the preparation of this work the authors used ChatGPT for tailored support for content organization and improving language and readability. After using this tool, the authors reviewed and edited the content as needed and take full responsibility for the content of the published article.

## CRediT authorship contribution statement

**Christiane Wesarg-Menzel:** Conceptualization, Data curation, Formal analysis, Investigation, Methodology, Writing – original draft. **Veronika Engert:** Conceptualization, Supervision, Writing – review & editing.

## Declaration of competing interest

The authors declare that they have no competing financial interests or personal relationships that could be perceived to have influenced the work reported in this paper.
