# Supplementary material for: Inducing stress, experiencing stress: Reflections and recommendations for the Trier Social Stress Test
Source: Compr Psychoneuroendocrinol. 2026 Jul 16;27:100368. doi: 10.1016/j.cpnec.2026.100368 (PMC13401023; doi:10.1016/j.cpnec.2026.100368)
Supplement: Multimedia component 1 [file mmc1.docx]

**Inducing Stress, Experiencing Stress:**

**Reflections and Recommendations for the Trier Social Stress Test**

**-**

**Supplementary Material**

(Wesarg-Menzel & Engert)

**Data acquisition and processing**

“To compile reports, we contacted almost 300 researchers via e-mail who published TSST-related work between 2023 and 2025, identified through PubMed and personal contacts. The following text was included in the e-mails:

--------------------------------------------------------------------------------------------------------------

Dear colleague,

For a special issue celebrating Clemens Kirschbaum’s contributions to Psychoneuroendocrinology, we are collecting personal experiences with the Trier Social Stress Test.

Given your expertise in conducting the TSST, we would be delighted if you could share any memorable or unusual experiences. These might include unexpected participant reactions, mishaps or challenges during implementation, setting-related issues, and reflections on what you might do differently in hindsight.

We plan to summarize these experiences anonymously, with the goal of generating practical and inspiring recommendations for both newcomers and seasoned TSST researchers.

We look forward to reading your contribution. If you wish, we would be happy to list you as a contributor in the acknowledgements.

With best regards,

Veronika Engert and Christiane Wesarg-Menzel

--------------------------------------------------------------------------------------------------------------

Approximately two weeks after the initial invitation, a reminder was sent to researchers who had not yet responded. In total, we received 32 responses via e-mail from 46 researchers. Please note that some responses were submitted jointly by multiple researchers, resulting in a higher number of contributors than responses.

All responses were saved in a text file and subsequently transferred to an Excel spreadsheet to facilitate the identification of overarching themes for data integration. The following categories were derived from the reports: overall evaluation of the TSST, TSST reactions (participants vs panel members), general advice, reflections, open questions, funny anecdotes, and words of honor for Clemens. Each report was assigned to one or more categories, depending on its content.

Coding was conducted manually by the first author without the use of a formal qualitative analysis framework. We aimed to incorporate all reports into the manuscript. Reports addressing similar topics were synthesized, whereas a small number of reports were omitted when their content could not be meaningfully integrated or when inclusion would have comprised the readability of the manuscript.

All reports are presented below in anonymized form (except for shared publications) and ordered chronologically according to their receipt (from earliest to latest). For reports originally written in German, an English translation is provided alongside the original text.

**Original reports**

1. “We conduct the TSST with children, some as young as 9 years of age. I imagine that Clemens has never had to deal with helping a child who wet his pants not feel bad. then there was the study in which we had the children bring their good friend to help them prepare for the speech. There were these two 9 year old boys who spent the whole speech prep time telling each other "yo mama" jokes (yo mama so old, she rode dinosaurs to school, yo mama so ugly she cracks the mirror) and cracking each other up. When it came time for the friend to leave and the target to give the speech the poor target was like a deer in the headlights.”
2. “Thank you for reaching out. We tested pregnant and early postpartum women, which may have influenced some of our experiences. However, almost all participants still completed the task. While some voiced clear discomfort, others did not seem to mind. Since this research was conducted a few years ago, I may be overlooking details, but I hope these reflections are useful.

We had two cases where participants refused the TSST after the explanation. One was a participant who declined because she had never applied for a job or taken part in a job interview before, which we had not anticipated given our sample (adults in their late 20s to 40s). I think it is valuable to plan ahead for refusals, especially in more vulnerable populations, so participants do not feel their visit was wasted. In our case, we provided an alternative task, though we did not include their data in the analyses.

We also had a few participants who became emotional or strongly disliked the TSST. For such cases, I believe it is important to have a clear protocol for both the researchers and jury on how to respond. For example, to stop the task early, especially in more vulnerable participants. Another challenge arose when a participant, though fluent in the testing language, struggled with the arithmetic task. She began confusing certain numbers, became frustrated, and nearly emotional. Maybe good to keep the native language of the participant in mind when testing.”

1. “Ich hatte während der TSST Testungen selbst keine unerwarteten Reaktionen von Prb.. Generell hatte ich aber das Gefühl, dass die Aufklärung zum Schluss extrem wichtig war, sodass die Prb. mit einem guten Gefühl nach Hause gehen konnten. Dazu gehörte sowohl das Setting (Kommittee nicht echt, Beleuchtung, Videoaufnahme) zu erklären und wieso das stresst als auch die Stressfaktoren (Unkontrollierbarkeit, Unvorhersehbarkeit, threat to the ego…) zu erläutern und auch im Notfall das Angebot zu machen, dass sie die Kommitteemitglieder einmal “normal” kennenlernen dürfen.”

[*translated*: During the TSST assessments, I did not personally observe any unexpected participant reactions. However, I had the impression that the debriefing at the end was extremely important to ensure that participants left with a positive feeling. This included explaining the experimental setup (e.g., that the committee was not real, the lighting, and the video recording) and why it induces stress, as well as discussing the relevant stress factors (uncontrollability, unpredictability, threat to the ego, etc.). When necessary, participants were also offered the opportunity to meet the committee members in a more informal setting afterward.]

1. “Thanks for asking! We did not have unusual experiences.”
2. “Thank you for your inquiry and for the great idea regarding summarizing personal experiences with the TSST. We have implemented the TSST in virtual reality in a school setting (with adolescents and young adults) and have had good experiences with it. Special features that occurred in our case: Three of our BPD patients dissociated during the TSST, which on the one hand could be handled by medical staff without further complications, but on the other hand shows that the TSST can also generate such a high level of tension in VR as in real life that these symptoms can occur. Our control condition did not seem to work quite as well and unfortunately also caused a relatively high level of stress in the test subjects. Further information on this can be found in the paper aVeRsive tension.”
3. “How fun! Well… I’ve used the TSST a few times. In one of the first stress studies I ran, I remember putting together my TSST team, but I was having a hard time getting research assistants (RAs) to run the sessions. Many found running the task to be rather stressful themselves, and didn’t want to conduct stress day sessions… until I found XY. XY THRIVED administering the TSST. Unlike my other RAs, who became nearly as distressed as the participants, XY took pride in a job well done – in this case, her ability to stress out her participants. She kept a tally of how many participants cried when she was working, and told the rest of the lab about the most intense sessions like they were badges of honor. Even more impressive – XY´s attitude about running the TSST was infectious. Pretty soon, all my RAs loved running stress sessions, and they hoped for (instead of dreaded) intense stress responses in their participants. Her mindset as a researcher running the TSST really helped motivate the lab.

I had also just finished piloting my dissertation research, which included a TSST, when covid happened. My university was back in action in the Fall of 2020, so I needed to figure out how to conduct the TSST in person, while keeping everyone 6 feet apart at all times. I tried a ton of modifications, using different spaces and table/camera set ups, and eventually landed on the following modifications: I couldn’t fit multiple panelists in the room, and have them 6 feet apart, in a way that felt natural, so I went down to one RA to administer the task. I also could not stress anyone out with a mask on – something about seeing people’s mouths not smiling seemed to matter. Instead, I had my RA wear a clear face shield.

Finally, one of my favorite things about the TSST is how many different factors it includes that helps it to induce stress for nearly everyone. However, it almost never works for the military veterans… I think in all my participants, I have maybe gotten a TSST induced rise in cortisol in all of two veterans – the other dozen or so were generally unfazed, or found the task fun!”

1. “What to consider when you perfom the TSST:

TSST-Procedure

-If you want to increase the stressfulness in a swiss version of the TSST, you might want to force subjects to count backwards in High German - though, at the same time you might challenge cross-national friendship...

-If you want high cortisol responses: let subjects perfom in a childish red plastic rain coat!

Research Team

-Do not cooperate with a colleague as a jury member that tries to make you laugh; that litteraly excerts bodily harm on you as a TSST jury member, especially in habituation studies with hundreds of testings in a row...

Subjects

-Do not place subjects (especially a male subject) near a heater when you want to draw blood: He might lose consciousness and bonk his head on the heater!

-Always keep cool as the lead experimenter: "Hey, young lady: there is blood spraying out of my arm!" (subject shouting and running back and forth the hallway spreading blood all over the place).

International Colleagues

-Don't be surprised when the TSST is just called "the TRIER" ("We did the Trier"). Although, this sounds really weird for us (former) Trier people.

Handling of Data

-Be aware: resulting data can be a dopey cow! (XY als Doktorand: "Die Daten sind ne dumme Kuh!")

-You should never postulate a directed hypothesis - it ALWAYS tuns out the other way round... (Schlussfolgerung von Clemens nach zahlreichen TSST-Experimenten)”

1. “Summary of the methodology:

The study sample comprised children and adolescents aged 8 to 16 years, allocated to three groups based on diagnostic status: autistic individuals without intellectual disability, individuals with Specific Learning Disorders (SLD), and a group of non-diagnosed peers.

The children’s version of the Trier Social Stress test (TSST-C, Buske-Kirschbaum et al., 1997) was used in this study to elicit a psychophysiological stress response in a controlled laboratory setting. The complete protocol consists of a 20-min session made of four 5-min components: (1) Baseline, (2) Preparation, (3) Public Speech, and (4) Recovery. Participants were instructed to prepare and deliver a five-minute speech about their ideal birthday party, aiming to outperform others, which introduced social comparison pressure. Participants were told their performance would be evaluated, but that competition rules prohibit judges from interacting with participants. At the end, a researcher debriefed the participants, explaining that the panel of judges was not truly judging them. This step was important to alleviate any discomfort induced by the social evaluative component of the task and to ensure ethical transparency in the experimental procedure.

In Study 1, behavioral (i.e., quality of the public speech) and subjective (i.e., valence, arousal, perception of competence, worries) components of anxiety were examined via the TSST-C. In Study 2, we also used electrocardiogram (ECG) measurements to track psychophysiological responses and autonomic changes across all phases of the protocol.

Anecdotes:

Most of the anecdotes refer to the target population (1. children and adolescents; 2. with neurodevelopmental conditions) which, despite displaying heterogeneous traits, shares common characteristics that make the TSST experience unique.

In this study, we used a wearable ECG device selected specifically for its suitability for children and adolescents, allowing for comfortable and minimally intrusive monitoring of cardiac activity throughout the TSST-C. However, in some cases, obtaining ECG measurements with autistic participants presented specific challenges due to heightened sensitivity to tactile stimuli. To facilitate electrode placement, the procedure had to be performed rapidly while engaging the participants in conversations about their personal interests as a form of distraction. Given their frequent curiosity about technology, it was also essential to provide clear and detailed explanations of each step involved in electrode placement and ECG recording.

In some cases, autistic participants experienced difficulties in formulating the speech about their ideal birthday party. When the experimenter inquired about the reason, participants explained that past experiences of social exclusion - such as having no peers attending their birthday parties - made the task particularly challenging.

Attention should be paid to the fact that children may move frequently or have difficulty remaining still, which can compromise the quality of ECG signals.

Similarly, the use of medications - commonly prescribed for autism - that could affect cardiac activity should be carefully considered, as they may influence the ECG recordings.

Across participants, irrespective of diagnostic group, the presence of the video camera was reported as the most discomfort-inducing aspect of the procedure. This likely reflects the heightened sense of social evaluation and self-consciousness induced by being recorded - an experience that differs from everyday situations, where such constant observation is uncommon. Consequently, this introduces a potential generalizability bias, as participants’ responses may be amplified compared with real-life public speeches.

Although participants were instructed to speak for the full five minutes until the panel indicated the end of the speech, they often concluded their presentations earlier due to running out of ideas.

Regarding the testing of children and adolescents without a diagnosis of autism, adolescents may sometimes show reduced collaboration, reflecting the developmental stage they are experiencing, which can affect engagement during experimental procedures.

We observed that adolescent female participants sometimes experienced embarrassment in the presence of male experimenters during electrode placement. This occurred even though we ensured full privacy and respect, by asking for parental written consent, participants’ verbal assent and explaining the procedure in details before doing it; did not require the removal of clothing; and positioned the electrodes in areas distant from intimate body regions. Indeed, particular attention should be paid to the sex of the experimenter when working with adolescent female participants, as having a male experimenter may negatively influence their comfort level, engagement, and responses during the procedure.

It should be noted that the TSST-C does not constitute a purely social stress task, but rather a performance-based stressor, as there is no direct interaction with the panel of judges, nor social exclusion. It is important to note that different patterns of social responses may be contingent on the type of stressor, thus autistic people might exhibit varying physiological reactivity to social interactions.

The fact that participants are unfamiliar with the judges and that they will not see them again may make the experience less frustrating; nonetheless, the task reliably elicits a moderate cardiac response also in participants who reported these types of thought, making it a suitable tool for studying psychophysiological stress.”

1. “We would be happy to contribute to this effort. My first thought was that our lab keeps a running log of advice for incoming research assistants who are learning how to be judges. You can view that advice here:

Wisdom on Administering the TSST-C

What strategies do you use to keep a straight face?

- I usually just zone out while the kids are talking and try not to listen to the story being told. Keeping a straight face has surprisingly not been too challenging, as most of the kids haven’t tried to tell funny stories at all. If you stay focused on writing notes frequently and maintaining eye contact with the child, it’s not too hard to keep a straight face. I’m usually so focused on watching the time and having my lines straight, I’m not really tempted to laugh.
- Zoning out helps and I usually just stare at the kids in the eyes while paying attention more to their mannerisms than what they are saying
- Think of dark, scary things, like the 2016 election.

What do you keep notes on during the story?

- I usually write most of my notes on behavior (i.e., fidgeting, avoiding eye contact, etc). Sometimes I’ll write down aspects of the story (if the participant is telling a particularly interesting or strange story).
- I’ll write notes on behavior, some interesting things of the story, sometimes just start scribbling/writing word cats
- I like to draw :)

What is the most unusual thing a participant has done during the TSST-C and how did you handle it?

- One of the participants I had really did not want to tell a story, and had to be prompted very frequently to continue telling the story. The participant stopped about 30 seconds in, and had to be prompted almost 10 times after that to continue speaking. Though the long pauses/silences can be awkward, they do effectively increase the stress, so I just waited it out.
- One participant I had refused to speak after being corrected once on the arithmetic portion. Zero eye contact and frozen body language. I kept the clock running until it was time to stop the stressor. I didn’t prompt him further because he understood the instructions, but was just paralyzed in fear.

Has the participant ever started to cry during the TSST? How did you handle that?

- I have never had a participant start to cry during the TSST.
- Yes, a participant was anxious during the speech portion so I gave them the space to cry. They continued their story regardless, so I continued with the protocol as normal (seems cold and cruel but se la vie). Obviously I reassured the participant during the debrief, which I think made him feel a little better.

Any other advice?

- Sometimes during the TSST, you’ll feel really bad for the participant. It’s hard to see them stressed out sometimes, and you’ll feel bad for being so “blunt” towards them. However, I always try to remind myself that in just a few minutes, I get to go back in and tell them it was all fake! They always smile and seem relieved, so just remember that the stressor is really temporary. Make sure to keep track of the time, and just focus on doing your lines properly.
- It’s important to maintain eye contact even if you aren’t in the Chair role for the TSST. Having eyes on the participant adds to their stress, so know your lines well enough were you can maintain as much eye contact as possible.
- Avoid nodding in agreement or even subconsciously, because it takes away from the facade. Think: robot.
- If you can’t help but giggle or smile, pretend you need to sneeze or pretend you have something on your upper lip so you can hide your mouth with your hand. Whatever you do, DO NOT turn to your lab partner or look at them when this happens.
- Speak slowly and clearly. Speaking too quickly gives the impression that you aren’t confident in what you’re saying, and takes away from suspense.
- If a participant begins saying the wrong number on the arithmetic portion (i.e. six hundred fif- I mean six hundred sixty!) then cut them off and call it an error. The point is to add to their stress, not be a fair referee.
- Whether you leave the story prompt with them until after debrief or take it as you leave after the arithmetic portion, make sure to keep this consistent throughout all participants.

We find that the hardest thing about being a TSST judge for pediatric participants (most of our work) is finding ways to not laugh... so a lot of the advice on remaining neutral throughout the story delivery focuses on strategies for keeping a straight face.”

1. “Ein mal ist ein Schüler bei einem TSST umgekippt, direkt in meine Arme, weil er so aufgerget war. Ein andermal hat ein Observer Child sich nach dem TSST übergeben. Und öfter haben Leute beim TSST geweint ... Ein Mal hatte ich einen Musiker als Probanden, der so übertrieben eine Show abgezogen hat, dass ich mich extrem bemühem musste, nicht zu lachen. Bei den Paar-TSSTs war es hitnerher so ,dass manche hintherher ihren Partner geneckt haben, weil die das so schelcht gemacht hätten ...“

[*translated*: One time, a student collapsed during a TSST, right into my arms, because he was so nervous. Another time, an observer child vomited after the TSST. And more often, people cried during the TSST ... One time, I had a musician as a participant who put on such an exaggerated performance that I had to make an extreme effort not to laugh. With the couple TSSTs, afterwards it was the case that some people teased their partner because they had done it so badly …]

1. “Trials and tribulations with the Trier Social Stress Test (TSST).

As a new graduate student, my prior research experience was, for lack of a better word, rather mundane—I was involved in simple behavioural tasks paired with surveys, with no social manipulation involved. Upon starting my MSc, my supervisor generously gave me the freedom to develop study ideas of my own. I presented several ideas, namely centred on influences of technology and AI, as these are the topics that I am largely interested in. As such, I was rather surprised when my project idea related to emotional states (i.e., stress) was one that was chosen. As eager as I was to conduct the study, it shortly dawned on me that I would, in fact, have to inflict psychosocial stress on undergraduate students (the demographic that makes up the most of our participant testing pool). After reconciling with the idea, I gathered a trustworthy team of undergraduate student researchers to assist me on this project. For our TSST, we followed the protocol outlined in a recent review by Narvaez Linares and colleagues (2020): a 5-minute preparatory period, a 5-minute job talk delivered to two judges and a camera, and a 5-minute challenging arithmetic task. The placebo (control) version included a 5-minute preparatory period, a 5-minute talk about a recent vacation or enjoyable book/movie (to a camera only, without judges), and a 5-minute simple arithmetic task. Below are two anecdotes written by my good student who acted as one of the judges in the TSST.

A withdrawal without warning

In all of the participants we ran, there was one who decided to leave the experiment midway through. While almost every participant exhibited some level of distress, this participant nearly broke down due to the stress caused by the TSST. Early in the verbal arithmetic task, the participant became noticeably choked up, stumbled on their words, and expressed that they could not answer the arithmetic problems. They then quickly explained that they could not continue and left the general experiment area with no follow-up. They were not heard from again until the experimenters contacted the participant a few hours later. Nearly every participant was visibly stressed at some point during the TSST portion of the experiment, but this was the only participant to actively remove themself from the study. The instructions given to this participant were no different than those given to the other individuals, leading us to believe that their experience was likely explainable by some internal factor. Experimenter follow-ups were in place to ensure no long-term negative effects would linger due to the study. The participant expressed that they were fine but did experience very high levels of stress due to the stoic and stone-faced nature of the interviewers. The interviewers were instructed to respond to any question with “please continue,” which left the participant in the dark as to any direction of what to do besides the initial instructions. Individuals with known anxiety disorders were screened out and not allowed to participate, highlighting this participant as an interesting case within the study.

Box Participant

There was one participant who was unlike the rest, and we colloquially referred to them as the “Box Participant.” While outliers are to be expected in research, this participant was not only an outlier because of their behaviour, but also because of the confidence and joy they brought to the experiment. When asked about their dream job, Box Participant outlined an entire philosophical view on how their current life situation existed in a box and how they wanted to break free from it. They constantly used hand gestures to draw a box in the air and motion as if they were destroying and rebuilding a new box. Box Participant, unlike most others, did not run out of things to say in the allotted five minutes for the dream job portion and had to be alerted and stopped at the time limit. They also did not specify their dream job at any point—just the philosophy that they carried. Most participants exhibited proficiency in only one of either the speech or arithmetic portion, but Box Participant continued their sprint by not making a single mistake and completing the challenging arithmetic task, where they were to verbally subtract 13 from 1022 for a 5-minute period, with surprising speed. Even after they left, the impact Box Participant created remained—they turned two stressful tasks into unproblematic chances to talk and have fun, something that no other participant was seemingly able to do.

Miscellaneous Reflection

Nearly every participant had a hallmark fidgety motion that they would perform once sufficiently stressed. This was usually just a tapping of the foot, swaying of the body, or twisting of their hair. It was interesting to note that although everyone experiences stress in different ways and different magnitudes, these fidgeting motions were largely consistent among participants. Similarly, nearly every participant presented efficiency in one half of the TSST but struggled with the other. For example, if a participant spoke confidently and without worry during the speech portion, they usually struggled in the arithmetic portion and vice versa. This quirk was especially interesting in cases where participants expended most of their energy giving their speech but were then informed that they still had an arithmetic task to perform. These participants appeared even more stressed out, likely because they thought all they would have to do was present a speech, leading them to believe they’d be able to exit the stressful social environment after they completed their interview. That break in their expectations could have been a contributing factor to their extra stress. This study was my first experience conducting research, leaving me with nearly no expectations as to what might happen. I never expected that my first time undergoing research would require me to stress people out, but it was a great opportunity to see the range of what different studies could look like. Noticing the varying habits of participants and how each individual was distinct yet still carried similar behaviours also caught my eye and sparked more research interest for me. Different variations of the study we conducted crowded my thoughts, like how changing one measure might lead to completely new results and how that might be relevant within the field of psychology in a different way. I continued working with my supervisor due to her insights and inspiring support, and I took what initially seemed like a unique research volunteer opportunity into something I want to pursue for years to come.”

1. “This sounds like a wonderful project to honor Kirschbaum’s significant contributions. I am not sure if this is what you are looking for; however, I am happy to share this reflection. The TSST remains the most potent and reliable laboratory stressor I have employed throughout my research career. Over the years, I have greatly valued the many thoughtful recommendations and methodological adaptations published to enhance its implementation. However, one aspect that receives far less attention is the impact of the TSST on the confederates themselves. Research assistants serving as TSST panel members frequently report experiencing their own distress in response to the procedure—often half-jokingly remarking, “You should be sampling my cortisol right now!” For this reason, an essential component of any TSST protocol should include dedicated time for the confederate to meet with the participant during the debriefing phase. This exchange allows both parties to process the experience, and it gives the confederate space to share whatever they need to—whether that be an apology, a reflection, or simply an acknowledgment that the stress induced by the TSST can be mutual”
2. “Hi and thank you for asking us to contribute anecdotes, I’m sure there are countless ones, but of course we always forget to write the funniest things down. Here is what xx and I came up with:

- One participant was “done” with the speech task after just one minute, stopped talking, and then—after ten seconds of silence—added, “Also, I’m very efficient.” (It was pretty hard for the panel of judges not to laugh)
- One participant “applied” to Deutsche Bahn, and the first attribute they mentioned was that they were “always on time.” …Again, hard not to laugh at that.
- One participant failed to get the cotton swab back into the salivette three times — they were probably quite stressed.
- The first participant is like the first pancake: Never turns out right.
- When you try to incorporate too many things (sensors, questionnaires, set-up changes), something always goes wrong — literally, always.
- It would be interesting to measure the cortisol levels of the panel/judges; The students helping with data collection are usually almost as stressed as the participants. (Although, somebody has probably done this before?!)“

1. “We had an experience once while performing the TSST protocol, when a patient start crying during the job interview. This specific patient had significant cortisol elevation and intraocular pressure elevation, showing that the TSST can simulate real psychological stress in some patients.”
2. “The TSST is a fantastic instrument to measure effects of stress in a realistic social setting. Indeed, the implementation of the TSST is not only stressful for the respective participants but also for the whole team conducting it! Doing the right things at exactly the right time - not to early, not to late - is also a great challenge for the whole study team. Adhering to the story of an unknown jury it also repeatedly influenced members of our team not to leave their offices for a longer time or to wait impatiently to use the public toilet to avoid meeting participants before taking part in the TSST jury, or to jump under the desk when a participant entered the wrong room to ask for further direction. Yet, these stressful hours completing the TSST are very much worth it given the thorough information we gain about stress reactivity using the TSST.”
3. “Please, see my reflections on the TSST use: First, some facts about our study (to give you a better overview of what we did):

- Please, see attached our study for a reference. Supplementary materials can be found [here](https://www.frontiersin.org/articles/10.3389/fpsyt.2025.1548287/full#supplementary-material).
- We used a partially-automated version of the test: We pre-recorded the interviewers giving instructions. The interviewers were present in a Zoom call. The experimenter (i.e., the person who welcomed the participant in the lab) was also present in the call but joined for real from another lab cubicle. Although in the original TSST, the experimenter is not present, we decided that they should join in order to answer questions from the participant, if there were any, and also to lightly interact with the pre-recorded interviewers, in order to increase the chances that the participant thought these were real. The participants were not informed that the interviewers were pre-recorded.
- The pre-recording of the interviewers was based on previous evidence from the attached studies that suggested that even such delivery of TSST (or another stress experiment) may increase stress in the participants.
- The Zoom version of the TSST was based on the [validation study](https://www.sciencedirect.com/science/article/pii/S0306453020305345?casa_token=Jx8frh7RQhAAAAAA:m9HrrPI3W0J0Veb4danagiKk54NAI5UMT1KZy-GKzqQ6tDQDwByADh6m7TOtaivhK0vemDOJK4E) of a Zoom protocol.
- We used a combination of two Dutch translations (in order to match our study perfectly) previously used in research (also attached - Starcke and Tollenaar).
- We also standardised the resting and recovery phase of the protocol, because we wanted to make sure that the participants watched the screen during the whole time (to video-record their facial expressions), and also to make sure that all the participants were doing the same thing. We picked a video of islands, because [it was previously suggested to be a relaxing activity](https://journals.plos.org/plosone/article?id=10.1371/journal.pone.0043571).
- We assessed physiological changes using the [VU-AMS system](https://vu-ams.nl/), and recorded video of the participants, but did not collect cortisol.

My observation during the study conduct (not sure if they are all relevant to TSST or to experiments in general):

- The TSST can be quite time- and resource-demanding. That is why we opted for the partially-automated version. It is of course not a perfect solution, but for us, it was quite a good trade-off between available resources and stress induction.
- The activity for the resting and the relaxation period is not standardised, and we were worried that participants could go immediately to their phones while waiting, which would not necessarily lead to them relaxing. Therefore, we searched for an activity which could be delivered in a standardised manner.
- The delivery of this partially-automated version was nevertheless quite difficult to program into our laboratory system. It was also difficult to make sure that the experiment always worked, because any update of the Microsoft or Zoom could destroy the balance of the programming.
- We had to stop the experiment with one participant because they became too stressed.
- Even in the partially-automated version, the experiment was very time- and resource-demanding. We also lost some of the participants, or at least part of their data, due to technological errors. Therefore, we did not manage to recruit all the participants we aimed to recruit (100) before the funding was over. That was of course also given by the fact that we had to wait for quite a few months before starting, due to the pandemic.
- Other than that, we did not observe anything unexpected or surprising, but I can understand that it might be due to the very unusual version of TSST that we used”

1. As previously mentioned, I am sending you an overview of the TSST anecdotes collected […]. I hope you will find something useful among them, wish you lots of enjoyment going through the material, and look forward to the corresponding publication.

- Eine Probandin hat als Berufswunsch Sexualtherapeutin angegeben und dann detailliert von ihren Berufserfahrungen und Vorstellungen berichtet…
- Bei Erhebung der Speichelproben sagt eine VP, dass sich eine Salivette so nass anfühlt (die wurde vorher schon der vorherigen VP abgegeben…)
- Bei wiederholtem Misslingen der Rechenaufgabe äußert eine Probandin Metakognitionen, die das Gremium unwillkürlich zum Lachen brachten: „2009… Ja, Mist, das habe ich eben schon verkackt, mal sehen, ob es jetzt gelingt: 1993! – Puh, OK, dann nächster Versuch, 2043, 2026, 2009, so, jetzt bin ich wieder an dieser schwierigen Stelle… 1994?“
- Person hat sich zunächst geweigert die Matheaufgabe zu machen
- Während der Instruktion entschied sich der Proband dazu, von dem Mikro wegzutreten und währenddessen seine Brille am Waschbecken zu putzen (in unserem TSST-Raum gibt es ein Waschbecken). Er war überall, aber nicht am Mirko
- Eine Probandin hatte kein Problem mit Schweigen und hat auf unsere Fragen nicht reagiert. Im ersten Teil haben wir uns dementsprechend 3 Minuten angeschwiegen
- Ein Proband war sehr groß (bestimmt 2m). Das Mikro war ihm viel zu klein. Er nahm das Mikro in die Hand. Zusammen mit dem Ständer. Wir im Gremium mussten etwas doll kichern…
- Person hat begonnen, Witze zu erzählen, „um die Stimmung aufzulockern“
- Das werde ich nie vergessen. Wir haben 7 min. über die optimale Zubereitung von Rosenkohl gesprochen.
- Ein Proband hat sich im Mathematik-Teil geweigert zu akzeptieren, dass seine Antwort falsch ist.

[translated:

- One participant stated that her desired profession was sex therapist and then went on to describe her professional experiences and aspirations in considerable detail...
- While collecting saliva samples, one participant remarked how wet a Salivette felt (it had previously been given to the preceding participant...).
- After repeatedly failing the arithmetic task, one participant voiced her metacognitions out loud, which involuntarily made the panel laugh: “2009... Right, damn, I already screwed that up before. Let's see if I can do it this time: 1993! – Phew, OK, next attempt: 2043, 2026, 2009, so, now I'm back at this difficult part again... 1994?”
- One participant initially refused to do the math task.
- During the instructions, the participant decided to step away from the microphone and clean his glasses at the sink instead (there is a sink in our TSST room). He was everywhere except at the microphone.
- One participant was completely comfortable with silence and did not respond to our questions. As a result, during the first part we spent three minutes sitting in silence together.
- One participant was very tall (certainly around 2 meters). The microphone was far too small for him. He picked up the microphone. Along with the stand. Those of us on the panel had a hard time suppressing our laughter.
- One participant started telling jokes “to lighten the mood.”
- I will never forget this. We spent seven minutes discussing the optimal way to prepare Brussels sprouts.
- One participant refused to accept during the mathematics section that his answer was incorrect.]

1. “The TSST has been a staple of my research for over twenty years now, and to this day, the paradigm lives up to its promise in eliciting robust stress responses. As a student assistant, back in the days (around 2000), I was trained as a TSST committee member, conducting hundreds of sessions stressing innocent study participants. Although we all know the various theories and models that explain how habituation works, I am afraid that I myself never really habituated to the TSST. Up to the last time I actively participated as a “stressor”, I always felt uneasy, stressed, and empathized with the poor participants. Still, not all encounters were unpleasant; there were a few participants whose performance stood out over the years. In an early study on the potentially protective effect of interpersonal touch, a male participant was, at the beginning of the TSST, invited by me to introduce himself and start his speech about why he would be the perfect fit for the job at hand. As the participants can pick the job they apply for themselves, it is always a bit of surprise what job exactly they are selecting for their introduction. This participant, though, did not pick the usual, but he told us that he is applying for clown school! Unfortunately (for us), he also started demonstrating his skills as a future clown. That was the one and only time when I really couldn´t contain myself and burst out laughing. It was a fun session, and believe it or not, this participant´s cortisol levels were markedly increased, despite the hilarious interaction we had (not sure, though, whether we actually used his data for the final analysis). Apart from this specific situation, doom and gloom prevailed, with participants becoming angry and running out of the room (not as many as one would think, though!), fainting (very few times, but it is good to have some first aid experience at hand), or freezing (to the extent that the TSST had to be ended prematurely). I am glad that nowadays, I can stay in the background and can let another generation of young stress researchers experience the TSST first-hand.

Let me thank Clemens Kirschbaum for his seminal work. Among his many achievements, creating the TSST and putting it out there for us to us is certainly one of the most enduring. I would like to mention that, in his honor, we named one of our lab rooms (the one that is dedicated to running TSSTs) the “Kirschbaum room” (the others are named “Selye”, “Cannon”, “McEwen”, etc.).”

1. **“**A brief history of failure: Stress protocols that we tested in pilot studies prior to developing the TSST, which did not work (and which, in reality, never received the names listed below)

*Trier Embarrassment Paradigm I*: An experimenter and a participant were in a room; the instruction to the participant was: “Stand on the table and recite a poem.”

*Trier Embarrassment Paradigm II*: An experimenter and a participant were in a room; the instruction to the participant was: “Stand on the table and sing a song.”

*Trier Learned Helplessness Paradigm:* An experimenter and a participant were in a room. Both sat at a table on which there were many different objects (a tennis ball, a pen, paper, a hole punch, etc.). The participant was constantly exposed to unpleasant noises played through headphones (the sound of a file being rubbed against guitar strings, chalk squeaking on a blackboard, etc.). The instruction to the participant was: “Find out what you have to do with the objects to make the experimenter turn off the noise.” Needless to say, there was no solution.

A reserve officer as perfect panel member? At the time of the first TSST studies, we had a student assistant who had been an officer in the German Armed Forces before starting his studies. He clearly enjoyed his role as a panel member (perhaps a little too much) and we were seriously concerned that our beautiful cortisol responses in the TSST might disappear as soon as he left our working group. Fortunately, this concern turned out to be unfounded.

Always ask about any unusual events before the lab visit. In a small twin study, we found extremely high baseline cortisol levels before the TSST in one pair. Only when we later asked for a possible explanation they did tell us that they had been involved in a car accident about an hour before the appointment, in which their car was completely destroyed.

In a small twin study, we found very high baseline cortisol levels in one pair prior to the TSST. It was only after we asked for possible explanations that they revealed they had been involved in a car accident approximately an hour before the appointment, in which their vehicle was badly damaged.

Always keep an examination table ready (or at least an air mattress). In a study conducted during a rather warm summer, participants were required to perform the TSST while having an IV cannula inserted. A considerable number reported feeling severely dizzy while standing in front of the panel. Although this phenomenon has occurred less frequently in subsequent studies, it still arises occasionally. Therefore, we always ensure that the TSST room includes a space where participants can lie down immediately if necessary.”

1. “Thank you for this opportunity to contribute to the special issue. I would like to share an interesting observation from my first TSST implementation in Southwest China in 2010. During this initial experience, I encountered a culturally specific challenge related to TSST. The local population in this region maintains a strong tradition of taking afternoon naps (siesta) following lunch. However, the manual of TSST suggested to carry out the test in the afternoon, which raised concerns about potential interference with our cortisol reactivity measurements in TSST, as napping during the typical CAR measurement window (30-60 minutes after awakening) could confound results. Although I was quite worried about this, the experiment results suggested the nap habits did not influence the cortisol reactivity induced by TSST. After that, I am quite convinced to carry TSST in the afternoon in China.”
2. “One of my first memories during my initial study with the TSST while I was doing my PhD is the unusual reaction of one participant. She was preparing for the job interview, and from the way she was preparing, the TSST team and the entire lab could guess what kind of job it was: She was an opera singer and prepared for the interview by warming up her voice with scales. I can't remember if her cortisol response in the TSST was particularly low… however, it wasn't easy for the panel to remain serious during the interview...”
3. We are deeply grateful for Dr. Clemens Kirschbaum's foundational contributions to stress research. Since its introduction in 1993, the Trier Social Stress Test (TSST) has remained the gold standard for inducing acute psychosocial stress. Its core elements—social-evaluative threat, uncontrollability, and the structured preparation-test-recovery sequence—have proven remarkably robust across decades of use. When we first began conducting TSST studies, our greatest uncertainty was sample size: How many participants are actually needed to reliably detect a stress response? Without clear guidance, we added participants incrementally until results stabilized—a post hoc and inefficient approach. To establish more principled guidance, we conducted a meta-analysis of existing TSST literature. This revealed distinct response magnitudes across different measures and a notably loose coupling between psychological and physiological systems (Gu et al., 2025; Gu et al., 2022). The fog finally lifted. We also strongly encourage fellow researchers to carefully consider the sample size required for each specific outcome and to adopt a comprehensive, multi-system approach when designing TSST studies.

Coordinating trained panelists became especially challenging when scaling up studies. After reviewing session videos and debriefing panelists, we noticed unintended variability in their facial expressions, posture, and nonverbal cues—subtle differences that introduced artificial heterogeneity into the stress induction. To address this, we developed a high-throughput TSST variant using pre-recorded, standardized evaluative audiences (Gu, 2022). Preliminary data suggest this approach maintains strong reliability and validity while reducing logistical burden and improving consistency and efficiency.”

1. “One summer day during testing for a sex and gender TSST study, a male participant chose to apply for the mock job interview as a gigolo. After a few minutes of comically selling himself and his services a sex worker , we intervened mid-way to switch sooner to the mathematics part with the following statement: “This interview has become inappropriate. We will now move onto the next task”. The participant apologized profusely while we the judges remained stoic in containing our laughter. Once complete, we decided to debrief the participant a little earlier as well since he felt guilty of crossing a line. In his defense, we do not specify the nature of the job people apply to in the TSST, so male escort is indeed legitimate if not somewhat unique in our studies.”
2. “We have appreciated the structure and format of the TSST and have utilized it in several laboratory studies. While we did not encounter any unusual participant reactions, some individuals experienced emotional distress or high levels of anxiety. However, they ultimately expressed gratitude for the opportunity to participate. Although the task challenged them, they were pleased to have successfully completed it.

I greatly value Clemens, not only for his work related to the TSST but also for his direct involvement in analyzing cortisol samples for multiple studies I conducted in Europe. He proactively reached out when several of my participants (police officers) exhibited cortisol levels significantly higher than ever previously observed, despite running repeated analyses to rule out measurement errors. This highlighted the upper limits of cortisol regulation among some law enforcement personnel and was a surprise to him at the time! It has been an honor to collaborate with him, and I sincerely appreciate his pioneering contributions to psychophysiology and psychoneuroendocrinology. Recognizing Clemens's contributions is indeed a wonderful idea.”

1. “Thank you for reaching out to me. The reason I remained silent in response to your previous emails is simply that there was nothing particularly noteworthy to report — except for one participant whose blood pressure suddenly dropped after the experiment began, forcing us to stop midway. Interestingly, the participant herself was also surprised by her own reaction afterward.

In my study, the participants were university students, and overall — especially the male participants — they seemed to enjoy the experiment. Some had to restrain themselves from giving spoilers to their friends, while others were eager to challenge their friends based on their performance.

I would also like to extend my special thanks to Clemens Kirschbaum, the originator of the experiment. When I reached out to him during the preparation phase with a question, he kindly replied within just a few hours — something I found incredibly valuable at the time.”

1. “My personal experience is that the TSST is extremely effective. As supervisor of the study I had to train several groups of students and when they practiced being panel member, I felt the nerves as mock-participant, despite the 20-year age gap. Once you start blushing as supervisor, you really feel embarrassed.
   A testament to long-lasting stress: Two years after having acted as a confederate for the panel, I (a student at the time) attended a university seminar where I noticed a younger classmate watch me from the corner of her eye. The seminar pertained to emotional memory, and after sharing my experience with the TSST, my classmate burst out: 'That is where I remember you from! I participated two years ago'. While she could not quite remember where she had met me, she shared that she had been nervous to approach and ask, feeling an unidentifiable discomfort when she saw me and heard me speak, even after all that time. Even after learning I was a student, the discomfort remained until we became better acquainted.

I remember one TSST session where at the end of the session, a building-wide alarm in the laboratory went off. We were as confused as the participant, though all panel members tried to stay in our role as best as we could, instructing the participant to head back to the experimenter. A tip based on this experience would be to discuss how to (if to) stay in the confederate role when unexpected occurrences occur, and to what extent the role can be deviated from (and in what situations). Preparation for the panel included practicing for the event that a participant burst into tears, which also occurred, though feigning control is more difficult when the unexpected happens.

Additionally, when using students as panel members, the consideration needs to be made whether these panel members can be separated as much as possible from participating students for the duration of data-collection (in the case of longitudinal data collection or EMA). Student panel members reported that they, at times, saw their participants (fellow students) from the day(s) before in the university building, and tried to hide as or avoid them as best as possible. One does not want to create the situation where panel members are insufficiently/less anxiety-inducing because they are a) already familiar to participants or b) because they are unmasked as being fellow students during the data collection period.

On a positive note, performing the TSST is not only possible with Bachelor students as confederates (in terms of sufficient stress induction and project management), but it was also considered to be a very enjoyable experience for the students. So, one does not need to shy away from this group when considering panel members if one has reservations about this.”

1. “Wir haben intern noch einmal gesprochen, können aber leider keine wirklichen Anekdoten beisteuern. Einzig dieser bereits veröffentlichte Interviewausschnitt wäre eine Möglichkeit:

[translated: We spoke internally once again, but unfortunately we cannot really contribute any anecdotes. The only possibility would be this interview excerpt that has already been published:]

“And what was horrible for me was that test day. […] This stress test. I thought it was extremely hard, but also brilliantly good. I thought, what’s going on? It was done professionally. It couldn’t have been better. […] And I thought to myself, these people are either such narcissists or they were professional actors.” (P05, male, 52 years) from Öznur et al., 2025

1. “The TSST is indeed a remarkable tool, and I am glad to share a funny experience.

I used the TSST in 2023 as part of a study investigating the effects of diaphragmatic breathing on acute stress recovery. While we had many compliant (and stressed) participants, one particular session stands out as a vivid reminder of the test’s *bidirectional* nature.

I was serving as the lead experimenter for the TSST committee that morning, wearing the obligatory lab coat and equipped with my best neutral academic demeanor. The participant was a 26-year-old male health professions student who greeted me with a bright, enthusiastic smile and remarkable energy. Undeterred, I delivered the standard instructions. When asked whether everything was clear, he responded with another full display of teeth, and I left him to prepare.

Ten minutes later, I re-entered the room with my colleague XY. This is a crucial detail: Maria and I share an almost telepathic sense of humor—the kind that makes one silently struggle to suppress laughter in serious settings. She is, therefore, arguably the worst possible colleague to co-administer a psychosocial stress induction protocol with.

We sat down at the committee desk. XY positioned the video camera and switched it on. At that exact moment, the participant’s eyes lit up unmistakably—he had found his stage.

We invited him to begin. With undiminished enthusiasm, he launched into a passionate monologue explaining why he was the ideal candidate to become a physician, describing his childhood aspirations and his profound vocation for caring for others.

However, after roughly two minutes, he reached his conclusion. Then, unexpectedly, he struck a static, almost mannequin-like pose, turned directly toward the camera, and proceeded to ignore us entirely.

Following protocol, I waited approximately ten seconds before reminding him that he still had time remaining. He replied with something akin to, “Thank you, I’m fine,” and remained motionless, continuing to smile directly at the camera. I repeated the prompt, clarifying that he was required to fill the full five minutes. Once again, politely but firmly, he declined, maintaining his fixed pose and expression.

There was something oddly compelling about his complete indifference to the evaluative presence of the committee. XY and I then endured what felt like two extraordinarily long minutes, maintaining our neutral expressions while facing his immobile, radiant smile. Our only relief came from observing the continuous physiological data on the monitor.

The transition to the arithmetic task felt like a reprieve for our own composure. It served as a powerful reminder that the TSST operates in both directions—effectively eliciting stress not only in participants, but sometimes in those administering it as well.”

1. “Ich habe eine kurze Anekdote aus meiner frühen TSST-Zeit am MPI, als wir einen Radiomoderatoren als Probanden hatten und er es sich zu seinem erklärten Ziel gemacht hat, uns zum Lachen zu bringen, dann aber mit der Zeit so sehr verunsichert war, dass er fast abgebrochen hätte. Ansonsten gibt es natürlich noch unzählige witzige Situationen aus den Hiwi-Einarbeitungen, bei denen wir uns lustige Berufe und Charaktere überlegt haben, um die Hiwis ein bisschen zu challengen (z.B. ein Imker, der extra Tanzen gelernt hat, um über Tänze mit seinen Bienen kommunizieren zu können und den TSST als Vortanzen nutzte).”

[translated: I have a short anecdote from my early TSST days at the MPI, when we had a radio presenter as a participant. He had made it his stated goal to make us laugh, but over time he became so insecure that he almost dropped out.

Apart from that, there are of course countless funny situations from training student assistants, during which we came up with amusing professions and characters to challenge them a little (for example, a beekeeper who had learned to dance specifically so that he could communicate with his bees through dance and used the TSST as an opportunity to give a dance performance).]

1. “So as a student researcher, I was sitting on the TSST panel in a twin study. Both twins were always tested on the same day, right after each other. Twin 1 steps in, does ok in job interview part, tells us he studies math. Then the mental arithmetic: without a single error, he reached 0 in 3 minutes and 47 seconds. We told him he could go. His twin brother was up next, and does exactly the same thing…. They were monozygous of course.”
2. “The recovery speed and extent of salivary cortisol after the TSST may be a good indicator, but this requires an increased collection density.

The collection of salivary cortisol itself is a disturbance, so it would be ideal to have wearable devices for collecting and analyzing cortisol in sweat.

Positive emotional responses after the TSST seem to be minimal, and this was later abandoned.

Some participants reported subjectively feeling not nervous, but their physiological responses might have been stronger.”

1. “I was always mega nervous during the TSST because it was happening in German. We didn't have any wild stories, unless it was something to do with what they were saying and I didn't understand ;)”
